# Incidence of Wound Infection After Abdominoplasty: A Retrospective Observational Study

**DOI:** 10.7759/cureus.108344

**Published:** 2026-05-06

**Authors:** Salman B AlNajjar, Suja AlZayer, Isa AlNajjar, Dalal A Yusuf

**Affiliations:** 1 Plastic Surgery, Salmaniya Medical Complex, Manama, BHR; 2 General Surgery, Salmaniya Medical Complex, Manama, BHR; 3 Family Medicine, Salmaniya Medical Complex, Manama, BHR

**Keywords:** abdominoplasty, plastic surgery, post operative complications, wound healing, wound infection

## Abstract

Aim

Abdominoplasty is a popular aesthetic and reconstructive procedure aimed at contouring the abdomen, addressing diastasis, and removing excess abdominal skin. Although it is generally safe, the incidence of wound infection remains a significant concern. Wound infections can increase medical costs, impact patients’ quality of life, and lead to further complications, including the need for reoperation. This retrospective questionnaire-based study investigates the incidence and associated symptoms of wound infection after abdominoplasty at Salmaniya Medical Complex.

Objective

To determine the incidence rate and symptoms associated with wound infection following abdominoplasty and to propose strategies for prevention and management.

Methods

A retrospective observational study was conducted on a total of 115 patients who underwent abdominoplasty between January 2022 and January 2024. Of these, 86 patients (74.8%) met the inclusion criteria and were included in the analysis. A modified Bluebelle wound healing questionnaire was utilized to categorize infection severity. The questionnaire included the following parameters: erythema, swelling, discharge, pain, warmth, wound odor, dehiscence, and fever. Each parameter had a scoring system of 0-3, with 0 being normal, 1 being mild, 2 being moderate, and 3 being severe, assessed subjectively according to the patient’s response. The total scores were then categorized as follows: 0-4, normal healing; 5-9, mild infection; 10-15, moderate infection; and >15, severe infection.

Results

The patient population can be summarized into three age groups. Patients aged 29-39 years made up 38.4% (n = 33) of the study population. The second group, aged 40-49 years, represented the highest percentage of the patient population at 43.0% (n = 37), and patients above 50 years old made up 18.6% (n = 16) of the total population. The mean age was 42 years, with a standard deviation of 8.45 years.

Of the 115 patients initially considered, 86 patients (74.8%) met the inclusion criteria and were included in the analysis. Of these 86 patients, 69 patients (80.2%), representing the majority of the population, had normal wound healing. Sixteen patients (18.6%) experienced mild infection, and upon follow-up questioning, all 16 patients improved with oral antibiotics without any further intervention. Only one patient (1.2%) experienced moderate infection, which resolved with oral antibiotics and local wound care. No patients were recorded as having severe infection (score >15).

Conclusion

The incidence of wound infection at Salmaniya Medical Complex after abdominoplasty was 18.6% for mild infection and 1.2% for moderate wound infection, for a total of 19.8%, which is in line with the literature review. Pain was the most common symptom, followed by redness and swelling. Postoperative monitoring of these signs and symptoms is warranted. Furthermore, targeted management of symptoms such as fever, discharge, and warmth may significantly reduce wound infection risks after abdominoplasty. Preoperative measures such as weight management and smoking cessation, combined with standardized wound care protocols, are recommended to improve outcomes.

Based on the data, localized wound symptoms were common, but serious complications were rare, reflecting a positive trend in wound healing and management within our center.

## Introduction

Abdominoplasty, also known as tummy tuck surgery, is a popular aesthetic and reconstructive procedure aimed at contouring the abdomen, addressing diastasis, removing excess abdominal skin, and strengthening the abdominal wall [1]. Since the 1960s, many authors have proposed different surgical techniques resulting in horizontal, vertical, or a combination of both scars [2]. Generally speaking, the surgery has a high satisfaction rate [3]. Although this procedure is typically safe, the incidence of wound infection remains a significant concern. Alongside the increase in bariatric surgeries, additional body-contouring procedures are being performed [4], and abdominoplasty has become increasingly popular in improving patients’ postoperative satisfaction [5]. Wound infections can occur with various procedures and can cause significant morbidity, especially in elective cases. Wound infections can lead to further complications, including reoperations, which can affect the patient’s quality of life and result in higher medical costs [6].

The objective of this study is to determine the incidence of postoperative wound infection in patients undergoing abdominoplasty at Salmaniya Medical Complex and to evaluate associated symptoms that may predict the severity of infection. Infection severity was assessed using a modified Bluebelle Wound Healing Questionnaire, and symptoms were analyzed to identify those associated with increased infection risk.

## Materials and methods

**Figure 1:**
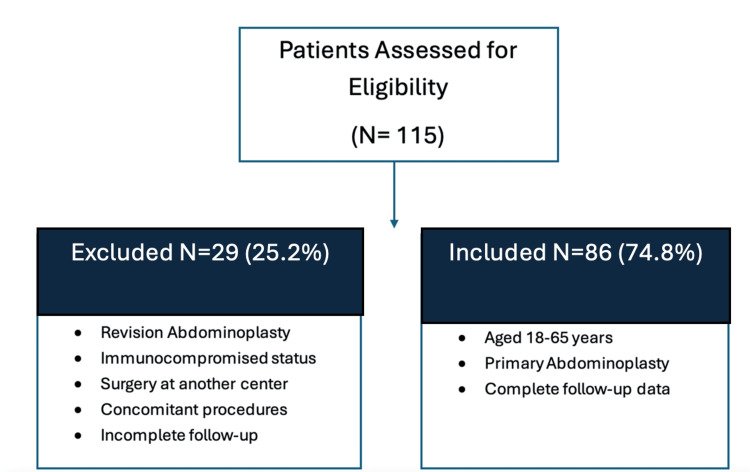
Inclusion and exclusion criteria.

A retrospective observational study was conducted on a total of 115 patients who underwent abdominoplasty between January 2022 and January 2024. However, only 86 (74.8%) patients met the inclusion criteria and were included in the analysis. Patients meeting the inclusion criteria were added to the study according to the flowchart in Figure 1; this included patients aged 18-65 years who underwent primary abdominoplasty with no concomitant procedures and had complete follow-up at our center. Conversely, patients undergoing revision abdominoplasty, those with an underlying immunocompromised status, those who underwent multiple procedures in the same setting, and those with incomplete follow-up were not included in the study.

A modified Bluebelle Wound Healing Questionnaire, which was developed for patient or observer completion, was used to categorize infection severity. The Bluebelle questionnaire is acceptable, reliable, and valid for post-discharge patient or observer assessment of surgical site infections in closed primary wounds [7]. The questionnaire asked patients to assess the following parameters: erythema, swelling, discharge, pain, warmth, wound odor, dehiscence, and fever. Each parameter was scored on a scale of 0-3, with 0 being normal, 1 being mild, 2 being moderate, and 3 being severe, assessed subjectively according to the patient’s response. The total scores were then analyzed and categorized as follows: 0-4, normal healing; 5-9, mild infection; 10-15, moderate infection; and >15, severe infection.

Data analysis was performed using IBM SPSS Statistics for Windows, version 30.0. ANOVA was used for symptom scores across severity categories, and the Kruskal-Wallis test was employed to compare non-normally distributed variables across patient groups. The p-value was set at <0.05.

## Results

From Table 1, it can be noted that the patient population was divided into three age groups: 29-39 years old (N = 33) comprised 38.4% of the study population, 40-49 years old (N = 37) comprised 43%, and age 50 and older (N = 16) comprised 18.6%. The mean age was 42 years old with a standard deviation of 8.45 years of age. 

**Table 1:** Patient age distribution. Values are presented as number (%).

Age group (years)	n	%
29-39	33	38.4
40-49	37	43
≥50	16	18.6
Total	86	100

**Figure 2:**
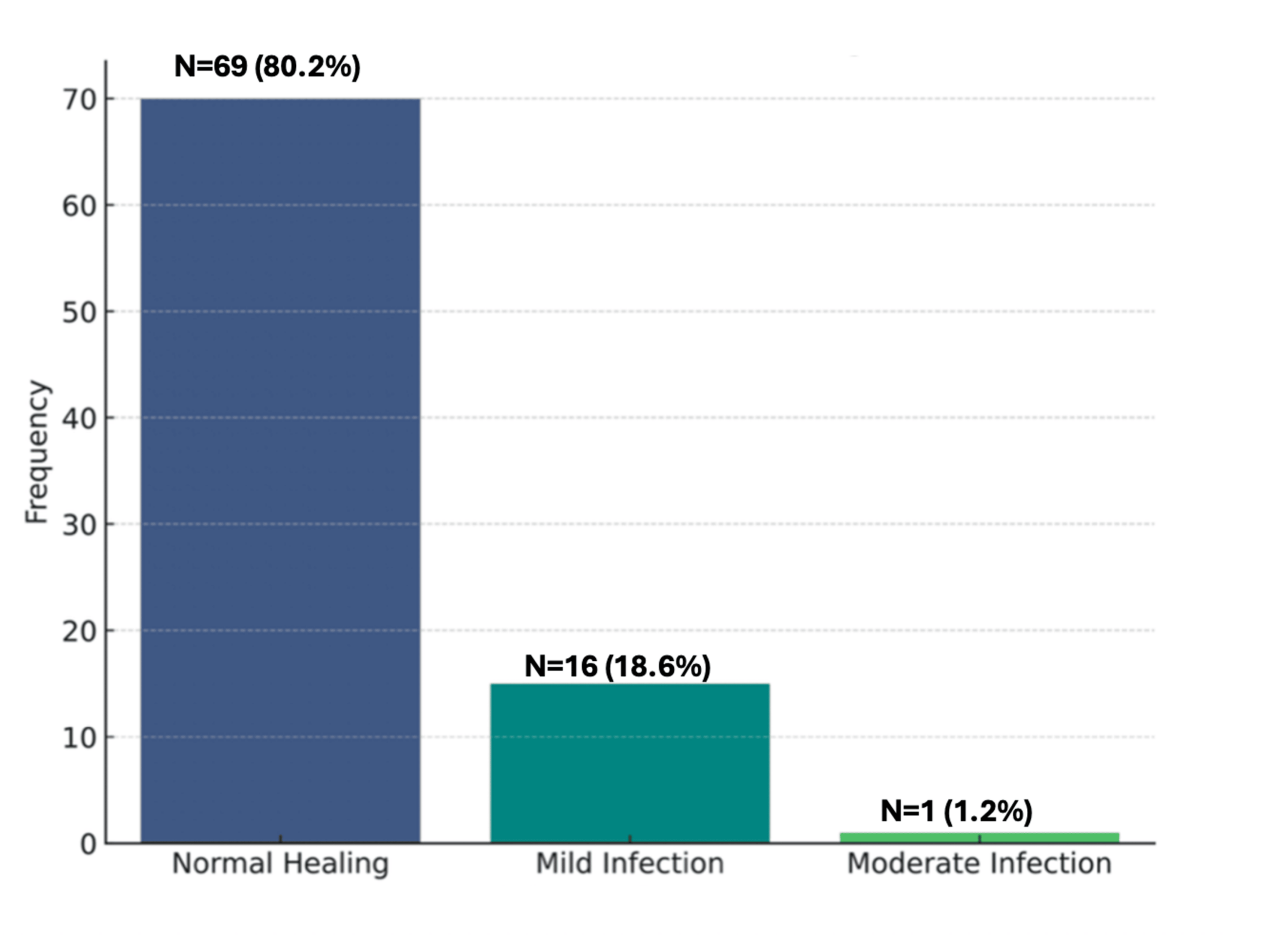
Frequency of wound infection categories.

Of the 115 patients initially considered, 86 (74.8%) met the inclusion criteria and were included in the analysis. As shown in Figure 2, of these 86 patients, 69 (80.2%) reported normal wound healing. A total of 16 patients (18.6%) experienced mild infection; upon follow-up questioning, all of these infections resolved with oral antibiotics without the need for any further action. Only one patient (1.2%) experienced a moderate infection, which resolved with oral antibiotics and local wound care. No patients reported severe infection.

**Figure 3:**
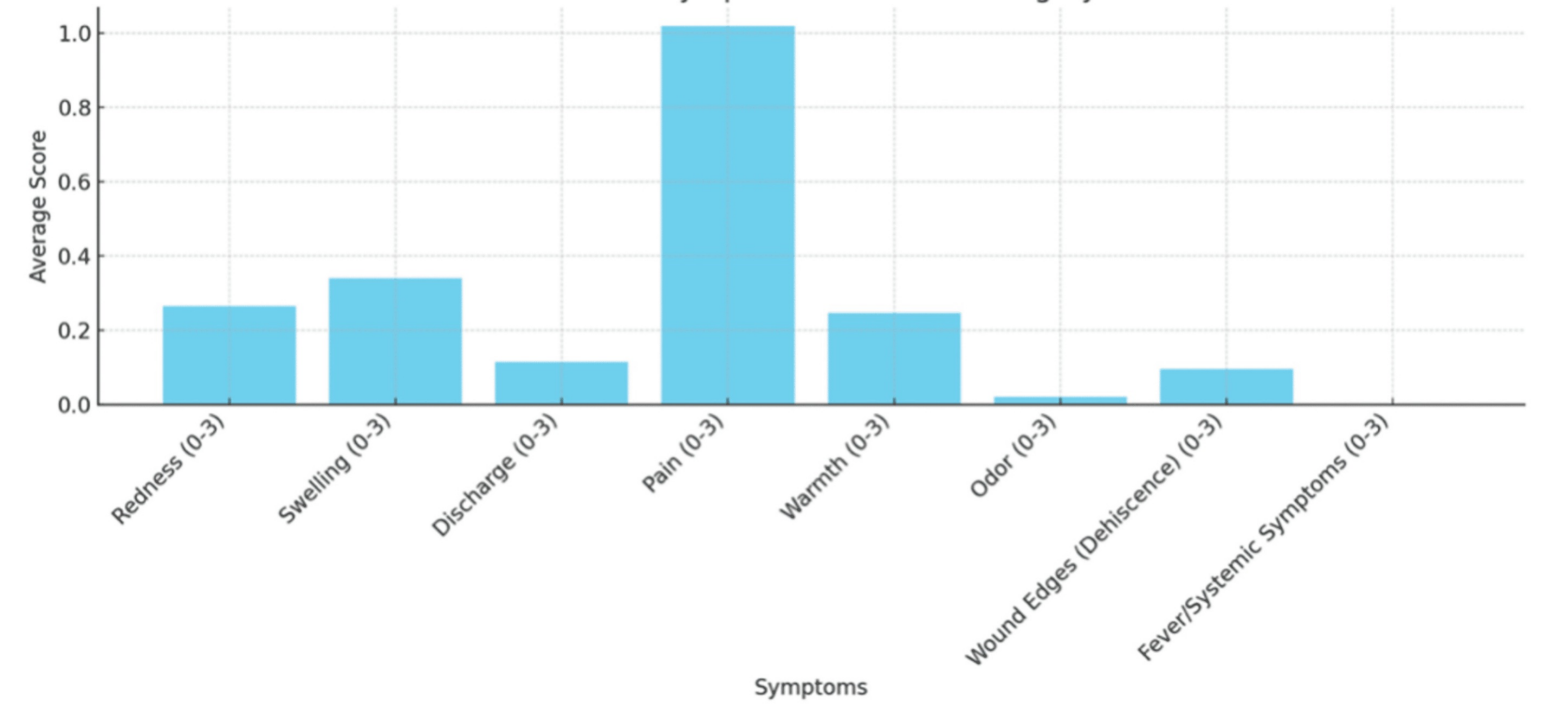
Average symptom scores in patients with normal wound healing. The following bar chart shows the average score for each of the listed symptoms in patients with normal wound healing.

**Figure 4:**
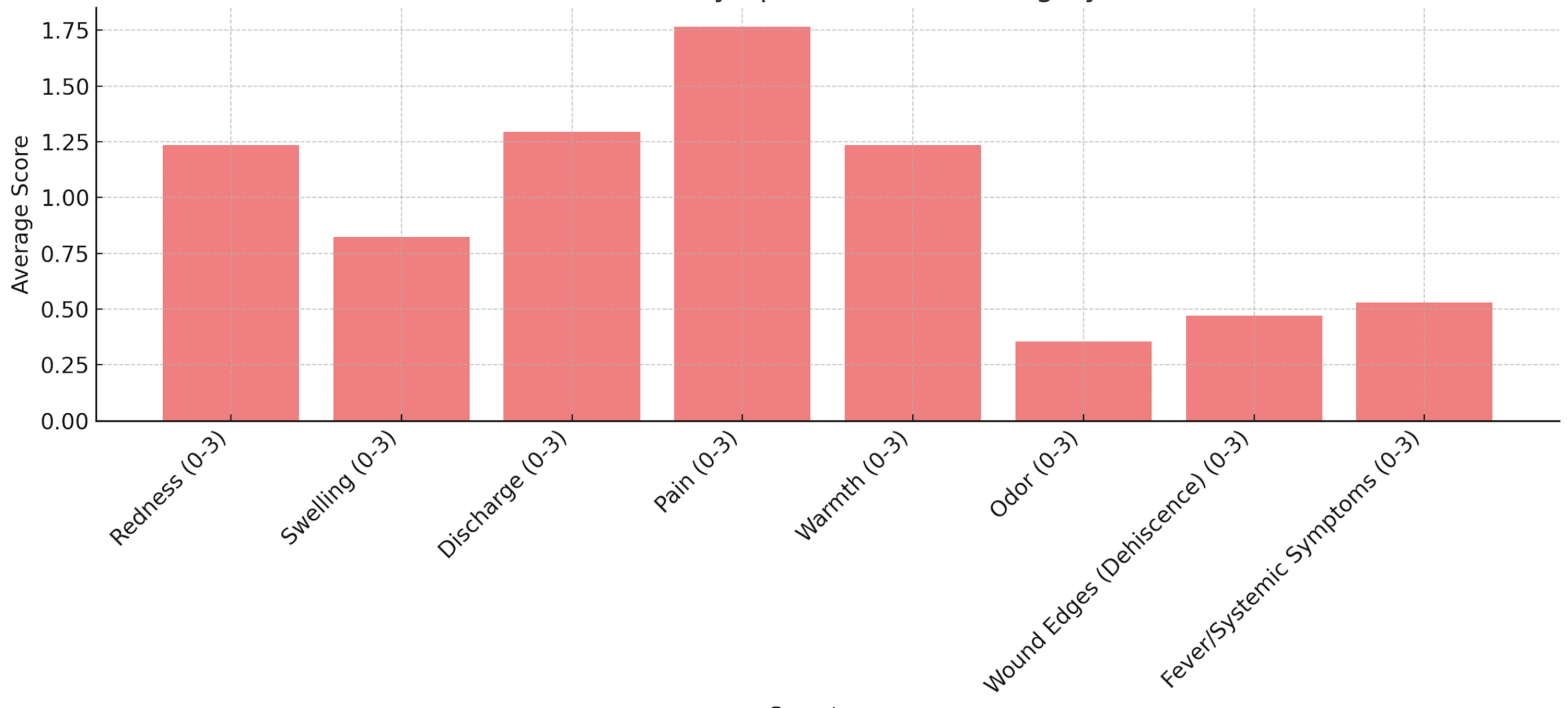
Average symptom scores in patients with mild infection. The following bar chart shows the average score for each symptom in patients with mild infection.

Descriptive analysis (Table 2) revealed a stepwise increase in mean symptom scores from normal healing (mean, 2.25) to mild infection (mean, 8.33) and moderate infection (mean, 12.0), supporting a clear gradient of clinical severity. This trend is visually reflected in Figures 3-4, where patients with mild infection exhibited higher average symptom scores across all domains compared with those with normal healing, particularly for pain, discharge, and redness. Collectively, these findings suggest that most clinical symptoms are reliable indicators of wound infection severity following abdominoplasty, with the exception of swelling, which did not show a consistent statistical association.

**Table 2:** Analysis of results. The table shows the mean, median, and range of the results.

Group	Mean	Median	Range
Normal healing	2.25	2	4
Mild infection	8.33	8	4
Moderate infection	12	12	0

Further analysis of symptom severity across wound healing categories demonstrated clear statistically significant differences. On ANOVA testing (Table 3), all assessed symptoms, including redness, swelling, discharge, pain, warmth, odor, wound dehiscence, and systemic complaints, showed significant variation between groups (p < 0.05), with redness (F = 21.13), discharge (F = 22.29), and warmth (F = 21.89) demonstrating particularly strong associations. These findings were corroborated by the Kruskal-Wallis test (Table 4), where all symptoms except swelling (H = 4.93, p = 0.82) remained statistically significant, further confirming a consistent relationship between increasing symptom burden and worsening infection severity.

**Table 3:** ANOVA test results. The table shows ANOVA test results with their respective significance values.

Symptom	F-value	p-value	Significance
Redness	21.13	<0.001	Significant
Swelling	3.23	0.04	Significant
Discharge	22.29	<0.001	Significant
Pain	6.93	<0.001	Significant
Warmth	21.89	<0.001	Significant
Odor	19.03	0.02	Significant
Wound edges/dehiscence	13.92	<0.001	Significant
Fever/systemic complaints	26.94	<0.001	Significant

**Table 4:** Kruskal-Wallis test results.

Symptom	H-value	p-value	Significance
Redness	26.95	<0.001	Significant
Swelling	4.93	0.082	Not significant
Discharge	24.87	<0.001	Significant
Pain	10.77	0.01	Significant
Warmth	22.49	<0.001	Significant
Odor	21.79	0.02	Significant
Wound edges/dehiscence	15.91	<0.001	Significant
Fever/systemic complaints	35.26	<0.001	Significant

## Discussion

A literature review highlights variable infection rates after abdominoplasty. Winocour J et al. reported an incidence of 1.4% [8], while Grieco M et al. observed a rate of 8% [9]. In contrast, Simon S et al. reported a significantly higher rate of 20% [10], while Neaman KC and Hansen JE reported that small-wound dehiscence, neuropathic pain, and minor cellulitis occurred in 26.7% of patients [11]. Identified risk factors include higher body mass index, smoking, prolonged operative time, and extended drain placement. The absence of severe infection cases in our study may reflect effective postoperative care [12]. However, the presence of mild and moderate infections suggests the need for enhanced monitoring and preventive measures.

It can be seen from Figure 3 that pain was the most common symptom in the normal healing group, consistent with findings in the literature showing that patients frequently report pain following abdominoplasty [13]. Evidence suggests that this pain can be most effectively managed with an intraoperative transversus abdominis plane (TAP) block [14] or a postoperative opioid infusion pump [15]. However, the TAP block is not performed routinely at our center. Similarly, as shown in Figure 4, pain remained the most common complaint in the mild infection group, which is expected given the combined effects of postoperative discomfort and localized infection.

A causal relationship exists between infection and pain [16], mainly due to manual manipulation of tissue and dissection followed by localized infection, which releases pro-inflammatory mediators resulting in increased pain perception [16].

It can also be noted from Figure 4 that discharge, warmth, and redness, followed by systemic complaints, were prevalent in the mild infection patient population. These are cardinal signs of local inflammation, correlating with postoperative local tissue inflammation and postoperative infection [17].

Significant associations between fever, discharge, and higher infection severity underscore the importance of targeted antibiotic therapy and improved wound care protocols [18]. Prolonged operative time, obesity, and smoking [19] are common risk factors associated with a higher likelihood of infection, and these warrant proper patient selection.

It can also be noted from the results of the ANOVA test reported in Table 3 that all parameters had a p-value under 0.05, which was considered significant. However, since the ANOVA test assumes a normal distribution, the Kruskal-Wallis test was also conducted, as shown in Table 4. Similar results were reported across all categories except for swelling, which was not significant. It can be noted that even in patients with normal wound healing, swelling can be accepted postoperatively as long as the other parameters are within normal limits. Soft-tissue edema after abdominoplasty is a well-documented phenomenon that occurs due to the presence of dead space caused by dissection. It usually resolves on its own or warrants lymphatic massage for drainage and the constant use of pressure garments. The use of quilting sutures is also a well-documented technique [20].

Furthermore, it should be noted that the percentage of patients over 50 years old (n = 16, 18.6%) and the percentage of patients with mild infection (n = 16, 18.6%) were both 18.6%, but this is coincidental. The patients in each infection group were of different ages.

Recommendations to minimize infection risks include preoperative weight management, smoking cessation [21], and standardized wound care protocols. Future studies with larger sample sizes and prospective designs are recommended to validate these findings.

Limitations of this study include the retrospective design, which restricts causal inference, and the single-center study site, which limits generalizability. Furthermore, the small sample size for the moderate infection category can be considered statistically underpowered. In addition, infection status was assessed according to the Bluebelle Wound Healing Questionnaire and was not confirmed by clinical examination or wound culture. Further studies may be conducted in which, following the questionnaire, patients are worked up to identify common organisms contributing to post-abdominoplasty wound infection. It is also important to mention that patients who did not respond to follow-up were not included in the analyses, which may have affected the results. Individual patient characteristics contributing to infection, such as operative time, BMI, and drain placement, were not analyzed in this study and may be contributing factors. Further studies may assess the association of these individual patient characteristics, whether intraoperative factors or underlying patient-specific medical conditions, with wound infection.

## Conclusions

The incidence of wound infection after abdominoplasty at Salmaniya Medical Complex (19.8% combined mild/moderate infection) is consistent with the global literature. While pain was the most frequently reported symptom, it is a nonspecific finding. Conversely, fever, discharge, and localized warmth served as significant predictors of infection severity. The lack of significance of swelling in statistical modeling suggests that it is a normal postoperative finding rather than a reliable indicator of pathology. Standardized postoperative monitoring and preoperative risk optimization, including smoking cessation and BMI reduction, remain cornerstones of reducing surgical site infections. Furthermore, targeted management of symptoms such as fever, discharge, and warmth may significantly reduce wound infection risks after abdominoplasty.

Based on the data, localized wound symptoms were fairly common, but serious complications were rare in this patient group, reflecting a positive trend in wound healing and management at the study site.
